# Characteristics and factors associated with mortality in tracheostomized patients with COVID-19: a retrospective cohort study in a hospital in Tacna, Peru

**DOI:** 10.17843/rpmesp.2023.404.12629

**Published:** 2023-12-18

**Authors:** Rodrigo Jesús Flores Palacios, Miguel Hueda Zavaleta, Andrés Guillermo Gutiérrez Avila, Juan Carlos Gómez de la Torre, Vicente Aleixandre Benites Zapata

**Affiliations:** 1 Daniel Alcides Carrión Hospital III, Tacna, Peru. Daniel Alcides Carrión Hospital III. Tacna Peru; 2 Postgraduate School, Private University of Tacna, Tacna, Peru. Private University of Tacna Postgraduate School Private University of Tacna Tacna Peru; 3 Diagnosis, treatment and research of infectious and tropical diseases, Private University of Tacna, Tacna, Peru. Private University of Tacna Diagnosis, treatment and research of infectious and tropical diseases Private University of Tacna Tacna Peru; 4 Roe Clinical Laboratory, Lima, Peru. Roe Clinical Laboratory Lima Peru; 5 San Ignacio de Loyola University, Research Unit for Health Evidence Generation and Synthesis, Lima, Peru. San Ignacio de Loyola University San Ignacio de Loyola University Research Unit for Health Evidence Generation and Synthesis Lima Peru

**Keywords:** Tracheostomy, Tracheotomy, Survival, SARS-CoV-2, COVID-19, Mortality, Peru, Risk Factors

## Abstract

**Objective:**

We aimed to describe the main demographic, clinical, laboratory and therapeutic characteristics and to identify whether they are associated with mortality in tracheostomized patients.

**Material and methods.:**

Retrospective cohort study in adult patients diagnosed with COVID-19, admitted to ICU (Intensive Care Unit) and requiring tracheostomy. Demographic, clinical, laboratory and treatment data were obtained from the medical records of patients admitted to Hospital III Daniel Alcides Carrión in Tacna. The Cox proportional hazards model was used for survival analysis and hazard ratios (HR) with their 95% confidence intervals (95%CI) were calculated.

**Results.:**

We evaluated 73 patients, 72.6% were men, the most common comorbidities were obesity (68.5%), type 2 diabetes mellitus (35.6%), and arterial hypertension (34.2%). Thirty-seven percent of the participants died during their stay at the ICU. The median time from intubation to tracheostomy and the duration of tracheostomy was 17 (RIC: 15-21) and 21 (RIC: 3-39) days, respectively. Multivariate analysis showed that the factors associated with mortality were procalcitonin > 0.50 ng/dL at the time of tracheostomy (HRa: 2.40 95%CI: 1.03-5.59) and a PaO2/FiO2 ratio less than or equal to 150 mmHg (HRa: 4.44 95%CI: 1.56-12.60).

**Conclusions.:**

The factors associated with mortality at the time of tracheostomy were procalcitonin > 0.50 ng/dL and a PaO2/FiO2 ratio less than or equal to 150 mmHg.

## INTRODUCTION

Coronavirus disease 2019 (COVID-19) has caused more than 5.4 million deaths worldwide and is the most important health crisis since the influenza pandemic of 1918 ^1^. As of July 24, 2023, nearly 4.5 million cases of COVID-19 have been diagnosed in Peru, with 221,203 deaths and a case fatality rate of 4.9% ^2^. Peru is the seventh country with the highest number of deaths in the world due to COVID and the third in Latin America ^3^.

Patients with acute respiratory distress syndrome (ARDS) and COVID-19 usually remain intubated for prolonged periods ^4^. Tracheostomy plays a fundamental role in the management of these patients when the intubation and mechanical ventilation time exceeds 14 days (prolonged mechanical ventilation) by allowing better airway management and facilitating the withdrawal of invasive ventilatory support. Tracheostomy has been associated with less need for deep sedation, shorter weaning time from the mechanical ventilator, shorter stay in the intensive care unit (ICU) and shorter hospital stay ^5^.

However, most ICU patients who are tracheostomy candidates have a higher risk of death due to the following characteristics: (a) ICU stay increases the risk of nosocomial infections ^6^; (b) prolonged mechanical ventilation, which requires longer sedation, analgesia and muscle relaxation, causes polyneuropathy and myopathy associated with immobility ^7^, (c) delirium upon withdrawal of sedation and analgesia; (d) the severe inflammatory process (cytokine storm) caused by COVID-19 directly affects gas exchange ^8^ and (e) the advanced age and presence of comorbidities that patients admitted to the ICU usually present ^9^.

Likewise, tracheostomy is a procedure that is not free of risks and potential complications such as hemorrhage, surgical site infection, pneumothorax and tracheal stenosis. So, due to all these conditions, tracheostomy is not always safe ^4^. Therefore, in order to improve clinical decision making and optimize results, the factors associated with higher mortality should be identified even if a patient with severe COVID-19 has the indication for tracheostomy, taking into account the high complexity and the accumulated mortality due to the baseline conditions of these patients.

So far, the factors related to mortality in critically ill patients tracheostomized due to COVID-19 are the following: high values of C-reactive protein (CRP) at the time of tracheostomy ^10^, higher inspired oxygen fraction (FiO_2_) requirement ^11^ and early timing of tracheostomy ^12^. Unfortunately, most of this information comes from developed countries and does not reflect the reality of Peru, a country with a fragile health system. In this context, critical illness due to COVID-19 was more frequent, aggressive and fatal ^13^, since after the three waves of this disease, the number of respiratory critical patients and tracheostomized patients increased, with a mortality rate of 32.9% in our ICU ^14^.

This study aimed to describe the demographic, clinical, laboratory and therapeutic characteristics as well as to identify predictors that allow timely identification of patients with COVID-19 who are at higher risk of death after percutaneous tracheostomy, since this group of patients have prolonged length of stay and high mortality rates ^15^.

**KEY MESSAGES**

Motivation for the study: Patients diagnosed with COVID-19 who are candidates for tracheostomy are complex cases with long hospital stay and high risk of death; the performance of this medical procedure in the best possible conditions is essential for their survival.

Main findings: The risk factors for death at the time of tracheostomy were low PaO_2_/FiO_2_ ratio (less than or equal to 150) and a procalcitonin level >0.5 ng/mL.

Implications: Early recognition of these risk factors may help to find the best time for the patient to undergo tracheostomy in a safer manner and with fewer complications.

## MATERIALS AND METHODS

### Study design

This was a retrospective cohort study. Data were collected between September and October 2022 and included patients with critical COVID-19, defined by the presence of ARDS, sepsis or septic shock ^16^, who were hospitalized at the Daniel Alcides Carrion Hospital in Tacna, from March 28, 2020 to March 1, 2022.

### Population

We analyzed 329 medical records from the Intelligent Health Service (ESSI), which is a software that contains digital medical records ^17^, including all patients hospitalized in the COVID-19 ICU (COVID-ICU) of the level III Daniel Alcides Carrión Hospital - EsSalud, located in Tacna, Peru, which has more than 120 beds for patients with COVID-19 and 14 beds in the ICU-COVID ^14^. Critical patients were treated according to the institutional protocols developed for patients with severe COVID-19; these protocols are constantly updated according to new scientific evidence.

We included patients over 18 years of age, admitted to the COVID-ICU, with a confirmed diagnosis of SARS-CoV-2 infection, severe respiratory failure due to COVID-19 and the need for invasive mechanical ventilation who, during their hospital stay, required percutaneous tracheostomy placement. All tracheostomies were performed percutaneously, by two ICU physicians, one in charge of the airway and the other in charge of the procedure itself. Patients admitted to the ICU for reasons other than COVID-19 pneumonia, patients with incomplete clinical/laboratory data and patients whose outcome was not death or decannulation were excluded. Patient death was corroborated by ESSI.

Although we analyzed 329 medical records, only 73 records of tracheostomized patients were included. Statistical power was calculated based on the study by Tang *et al*. ^26^, in which 73.3% of patients with early tracheostomy (≤14 days) died during follow-up, compared to a 42% mortality rate in those with late tracheostomy (>14 days). We considered an unexposed/exposed ratio of 1.30 (73/56). With these parameters, a confidence level of 95% and 73 participants in our sample, we obtained a statistical power of 76.5%.

### Study variables

The dependent variable was death during hospitalization (yes/no), with survivors being patients who were decannulated and subsequently discharged; this information was collected directly from the ESSI. The independent variables were age (years), sex (male/female). We considered comorbidities as dichotomous variables (yes/no), these were obesity, diabetes *mellitus*, arterial hypertension, and chronic kidney disease. The infection period was considered by years: 2020, 2021, and 2022. Vaccination (2 doses/3 doses). The time variables were: hospital stay (days), ICU stay (days), days with mechanical ventilation, days from intubation to tracheostomy, days of tracheostomy, and late tracheostomy was categorized as >14days (yes/no).

We obtained the following laboratory data: leukocytes (>12,000 cells/mm^3^), C-reactive protein (CRP>6 mg/dL), procalcitonin (procalcitonin> 0.5 ng/dL), PaO_2_/FiO_2_ (arterial oxygen pressure / inspired oxygen fraction) at the time of tracheostomy (≤150 mmHg), creatinine (>1.3 mg/dL). The ventilatory variables were: FiO_2_ (>35%), PEEP (positive end-expiratory pressure) (>8 cm H_2_O), tidal volume (>460 mL) and inspiratory pressure (15 cm H_2_O), peak pressure (>25 cm H_2_O), respiratory rate (>25 rpm). We included the following complications during hospitalization: superinfections, defined as clinical deterioration recorded and associated with a positive culture (yes/no), these were: bacteremia (yes/no), ventilator-associated pneumonia (yes/no), urinary tract infection (yes/no), septic shock (yes/no) and barotrauma (yes/no).

### Procedures

All patients hospitalized during the COVID-19 pandemic from March 28, 2020 to March 1, 2022 were included. Once identified, medical records were reviewed through the ESSI, information was collected on comorbidities, diagnoses, laboratory tests, procedures and outcome.

Data was collected by the researchers (RFP, MHZ and AGA) in a spreadsheet created in Microsoft Excel 2019; double data entry was performed to control inconsistencies. Demographic, clinical and laboratory characteristics were detailed at the time of the tracheostomy and additionally the treatment and its outcome during hospitalization (decannulation or death) were also collected.

The cases were followed during their stay in the critical care unit. The time of tracheostomy was considered to be time zero and the final time as the occurrence of death or discharge from the intensive care unit in decannulated patients.

### Statistical analysis

Data were imported into Stata® v17 (StataCorp., College Station, TX, USA). Comparisons were made between critically ill patients with tracheostomy who were successfully decannulated and those who died during their stay in the COVID-ICU. Categorical variables were expressed as frequencies and percentages, and numerical categorical variables as medians and interquartile ranges, as appropriate. All hypothesis tests were two-tailed, with a significance level of 0.05. Regarding the comparison of variables, the chi-square test or Fisher’s exact test (as appropriate) and the U-Mann-Whitney test were used for numerical variables, due to their non-normal distribution. In order to determine the factors associated with mortality at the time of tracheostomy, we used the Cox proportional hazards model, with the time variable being the days elapsed from hospitalization to death or discharge (in decannulated patients), to identify the crude and adjusted hazard ratios (HR) and their respective 95% confidence intervals (CI).

The variables that showed a statistically significant association in the bivariate analysis of mortality (p<0.05) were included in the crude analysis. The variables with a statistically significant association with mortality in the crude analysis (p<0.05) were included in the adjusted analysis. Proportionality assumptions were corroborated using Schoenfeld residuals. The collinearity of the variables included in the Cox regression was calculated using the variance inflation factor (VIF) with a cut-off point of 6.0. All the values of the variables included in the regression were acceptable [infection period (VIF=3.06), procalcitonin>0.5ng/dL (VIF=1.73), PaO_2_/FiO_2_ <150 mmHg (VIF=2.21), FiO_2_ >35% (VIF=1.98), and peak pressure >25 (VIF=3.33) [.

Finally, we described the survival of patients undergoing tracheostomy admitted due to COVID-19 using the Kaplan-Meier method. The log-rank test was used to evaluate differences between survival functions.

### Ethical Aspects

This research complies with the Helsinki norms for research on human beings. The research protocol was approved by the ethics committee of the Hospital III Daniel Alcides Carrión (CIEI-Tacna-N.º 036). This study was registered in the Health Research Projects Platform (PRISA) of the National Institute of Health with code EI00000003060. Due to the retrospective and observational nature of the study, informed consent was not requested and the data collected were confidential.

## RESULTS

Of the 329 critically ill patients hospitalized in the COVID-ICU, 83 (25.2%) underwent tracheostomy as part of their evolution, but 10 did not meet the inclusion criteria (one patient was admitted with a diagnosis other than COVID-19 pneumonia, 2 patients did not have the outcome of death or decannulation and 7 patients had incomplete clinical and laboratory data). We included 73 medical records of patients admitted to COVID-ICU with a diagnosis of SARS-CoV-2 infection, COVID-19 pneumonia and percutaneous tracheostomy carriers.

The median age was 59 (IQR: 52-66) years, 72.6% of the patients were male and only 6.8% were vaccinated against SARS-CoV-2. The most frequent comorbidities were obesity (68.4%), type 2 diabetes *mellitus* (35.6%) and arterial hypertension (34.2%).

The median ICU and mechanical ventilation (MV) length of stay was 32 (IQR: 24-41) and 31 (IQR: 24-43) days, respectively, the median tracheostomy time was 18 (IQR: 10-25) days (decannulation or death) and the time at which tracheostomy was performed was 17 (IQR: 15-20) days counted from the time of intubation.

Regarding laboratory tests, the median PaO_2_/FiO_2_ at the time of tracheostomy was 213 (IQR: 175-270), procalcitonin was 0.22 ng/mL (IQR: 0.13-0.40), CRP of 6.35 mg/dL (IQR: 3.2-12.1), and leukocytes of 11,950 cells/mL (8.810-15.510). Concerning mechanical ventilation variables, FiO_2_ at the time of tracheostomy was 30% (IQR: 28-35), PEEP (positive end-expiratory pressure) was 7 cm H_2_O (IQR: 6-8), inspiratory pressure was 15 cm H_2_O (IQR: 14-18) and the tidal volume was 466 mL (IQR: 406-509).

Regarding the treatment, 71 (97.3%) patients received antibiotics, 20 (27.4%) received antifungals and 9 (12.3%) received vasopressors at the time of tracheostomy. MV-associated pneumonia was found in 66 patients (90.4%), bacteremia in 14 (19.2%) and urinary tract infection in 9 (12.3%) patients. We found that 27 (37%) tracheostomized patients died during ICU stay and 46 (63%) were successfully decannulated and discharged (Table 1).

**Table 1:** Clinical, laboratory characteristics and ventilatory parameters of the study population and comparison between surviving and deceased decannulated patients.

Variable	Total n=73 (%)	Decannulated survivors n=46 (%)	Deceased n=27 (%)	p-value
Demographic characteristics				
Age ^a^	59 (52-66)	58.5 (49-65)	61 (52-66)	0.212^b^
Sex				
Male	53 (72.6)	34 (64.2)	19 (35.8)	0.743^c^
Female	20 (27.4)	12 (60.0)	8 (40.0)	
Clinical characteristics				
Comorbidities				
Yes	65 (89.0)	39 (60.0)	26 (40.0)	0.247^c^
No	8 (11.0)	7 (87.5)	1 (12.5)	
Obesity				
Yes	50 (68.5)	20 (58.0)	21 (42.0)	0.191^c^
No	23 (31.5)	17 (73.9)	6 (26.1)	
Diabetes *mellitus*				
Yes	26 (35.6)	15 (57.7)	11 (42.3)	0.484^c^
No	47 (64.4)	31 (65.9)	16 (34.1)	
Hypertension				
Yes	25 (34.3)	15 (60.0)	10 (40.0)	0.700^c^
No	48 (65.7)	31 (64.6)	17 (35.4)	
Chronic kidney disease				
Yes	3 (4.1)	2 (66.7)	1 (33.3)	0.693^d^
No	70 (95.9)	44 (62.8)	26 (37.2)	
Infection period				
2020	22 (30.1)	10 (21.7)	12 (44.4)	
2021	42 (57.5)	32 (69.6)	10 (37.0)	0.025^c^
2022	9 (12.4)	4 (8.7)	5 (18.6)	0.261^d^
SARS-CoV-2 Vaccination	5 (6.9)	2 (40.0)	3 (60.0)	
2 doses	2 (2.7)	0 (0.0)	2 (100.0)	
3 doses	3 (4.1)	2 (66.7)	1 (33.3)	
Days of hospital stay ^a^	41 (34-51)	47,5 (39-57)	34 (29-40)	<0.001^b^
Days of stay in ICU ^a^	32 (24-41)	32,5 (24-46)	27 (22-36)	0.172^b^
Days on mechanical ventilation ^a^	31 (24-43)	31,5 (23-45)	31 (27-38)	0.899^b^
Days from intubation to tracheostomy ^a^	17 (15-20)	16 (13-21)	17 (15-20)	0.791^b^
Tracheostomy days ^a^	18 (10-25)	19 (10.5-27.5)	13 (8-20)	0.087^b^
Late tracheostomy >14 days				
Yes	56 (76.7)	33 (58.9)	23 (41.1)	0.189^c^
No	17 (23.3)	13 (76.5)	4 (23.5)	
Laboratorial characteristics at the time of Tracheostomy				
Leucocytes >12,000 cel/mm^3^				
Yes	36 (49.3)	19 (52.8)	17 (47.2)	0.074^c^
No	37 (50.7)	27 (73.0)	10 (27.0)	
C-reactive protein >6 mg/dL				
Yes	33 (45.2)	17 (51.5)	16 (48.5)	0.065^c^
No	40 (54.8)	29 (72.5)	11 (27.5)	
Procalcitonin >0,5 ng/dL				
Yes	14 (19.2)	2 (14.3)	12 (85.7)	<0.001^c^
No	59 (80.8)	44 (74.6)	15 (25.4)	
PaO_2_/FiO_2_ <150 mmHg				
Yes	9 (12.3)	0 (0.0)	9 (100.0)	<0.001^c^
No	64 (87.7)	46 (71.8)	18 (28.2)	
Creatinine >1,3 mg/dL				
Yes	9 (12.3)	5 (55.6)	4 (44.4)	0.621^c^
No	64 (87.7)	41 (64.0)	23 (36.0)	
Ventilatory parameters at the time of tracheostomy				
FiO_2_ >35%				
Yes	27 (37.0)	11 (40.7)	16 (59.3)	0.003^c^
No	46 (63.0)	35 (76.1)	11 (23.9)	0.003^c^
PEEP >8 cm H_2_O				
Yes	28 (38.4)	16 (57.1)	12 (42.9)	0.412^c^
No	45 (61.6)	30 (66.7)	15 (33.3)	
Tidal volume >460 mL				
Yes	41 (56.2)	27 (65.9)	14 (34.1)	0.569^c^
No	32 (43.8)	19 (59.3)	13 (40.7)	
Inspiratory pressure >15 cm H_2_O				
Yes	48 (65.8)	28 (58.3)	20 (41.7)	0.251^c^
No	25 (34.2)	18 (72.0)	7 (28.0)	
Peak pressure >25 cm H_2_O				
Yes	28 (38.4)	12 (42.9)	16 (57.1)	0.005^b^
No	45 (61.6)	34 (75.5)	11 (24.5)	
Respiratory frequency >25rpm				
Yes	22 (30.1)	13 (59.1)	9 (40.9)	0.648^c^
No	51 (69.9)	33 (64.7)	18 (35.3)	
Complications				
Nosocomial infection				
Yes	64 (87.7)	39 (60.9)	25 (39.1)	0.701^d^
No	9 (12.3)	7 (77.7)	2 (33.3)	
Ventilator-associated pneumonia				
Yes	66 (90.4)	41 (62.1)	25 (37.9)	0.483^d^
No	7 (9.6)	5 (71.4)	2 (28.6)	
Bacteriemia				
Yes	14 (19.2)	10 (71.4)	4 (28.6)	0.344^d^
No	59 (80.8)	36 (61.0)	23 (39.0)	
UTI				
Yes	9 (12.3)	5 (55.6)	4 (44.4)	0.440^d^
No	64 (87.7)	41 (64.0)	23 (36.0)	
Septic shock				
Yes	63 (86.3)	42 (66.7)	21 (33.3)	0.098^d^
No	10 (13.7)	4 (40.0)	6 (60.0)	
Barotrauma				
Yes	14 (19.2)	7 (50.0)	7 (50.0)	0.262^c^
No	59 (80.8)	39 (66.1)	20 (33.9)	0.262^c^

a Median and interquartile range, ^b^ Mann-Whitney U test, ^c^ Chi-square test, ^d^ Fisher exact test.

ICU: intensive care unit; PaO_2_/FiO_2_: arterial oxygen pressure to inspired oxygen fraction (FiO_2_) ratio; PEEP: positive end-expiratory pressure; VAP: ventilator-associated pneumonia; UTI: urinary tract infection; rpm: respirations per minute.

No significant differences were found regarding the mortality in patients according to age group, sex, vaccination status, comorbidities, length of stay in the ICU, time on mechanical ventilation, time of tracheostomy, or according to the treatment. However, the time of tracheostomy was longer in those who achieved decannulation than in those who died (21 vs. 15 days; p=0.043). Likewise, PaO_2_/FiO_2_ was lower in the group of patients who died (p<0.001), while a procalcitonin value >0.5mg/dL, higher FiO_2_, inspiratory pressure and peak pressure at the time of tracheostomy were associated with a higher risk of death.

**Figure 1:**
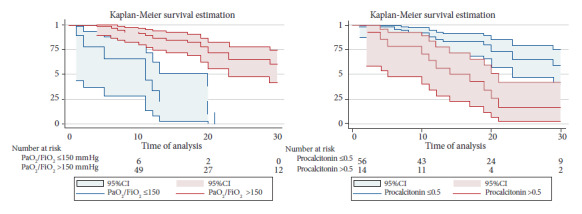
Kaplan-Meier survival curves according to PaO_2_/FiO_2_ and procalcitonin level, censored at 30 days**.**

Factors associated with mortality were evaluated by Cox proportional hazards regression. In the adjusted analysis, procalcitonin >0.50 at the time of tracheostomy was shown to be a factor associated with mortality with a aHR of 2.40 (95%CI: 1.03-2.59). Likewise, a PaO_2_/FiO_2_ level ≤150 mmHg was independently associated with higher mortality, with an aHR of 4.40 (95%CI: 1.56-12.60) (Table 2). Figure 1 presents the Kaplan-Meier survival curves for the variables PaO_2_/FiO_2_ (p<0.001) and procalcitonin (p<0.001).

**Table 2:** Cox regression analysis to evaluate predictors of mortality in tracheostomized patients hospitalized due to COVID-19.

Variable	cHR (95%CI)	p-value	aHR (95%CI)	p-value
Infection period				
2020	Ref.		-	
2021	0.52 (0.22-1.21)	0.130	-	
2022	1.13 (0.39-3.26)	0.816	-	
Procalcitonin >0,5 ng/dL				
Yes	3.76 (1.75-8.10)	0.001	2.41 (1.04-2.60)	0.005
No	Ref.		Ref.	
PaO_2_/FiO_2_ ≤150 mmHg				
Yes	7.60 (3.21-17.99)	<0.001	4.41 (1.57-12.61)	0.005
No	Ref.		Ref.	
FiO_2_ >35%				
Yes	2.02 (0.93-4.36)	0.075	-	-
No	Ref.		-	
Peak pressure >25 cmH_2_0				
Yes	2.40 (1.09-5.29)	0.030	1.31 (0.52-3.30)	0.569
No	Ref.		Ref.	

cHR: crude hazard ratio; aHR: adjusted hazard ratio; PaO_2_/FiO_2_: ratio of arterial oxygen pressure to inspired oxygen fraction (FiO_2_); PEEP: positive end-expiratory pressure. The variables included in the adjusted analysis were those that showed a statistically significant association with mortality in the crude analysis (p<0.05).

## DISCUSSION

We found that 36.9% of all patients who were tracheostomized by COVID-19 died. Furthermore, we were able to establish two risk factors for mortality, which are the procalcitonin value >0.5 ng/dL, and a low PaO_2_/FiO_2_ ratio (less than or equal to 150) at the time of tracheostomy.

Reported mortality rates in patients with tracheostomy and COVID-19 ranges from 15 to 66% ^18^^-^^21^. A meta-analysis of 5268 tracheostomized patients reported a pooled mortality of 22%; however, there was great heterogeneity among the included studies ^19^. It has also been reported that critically ill patients who underwent tracheostomy had lower mortality than those who were not tracheostomized, even in severely ill patients with APACHE II >17 ^18^^,^^21^. In Peru, the overall mortality due to ARDS associated with COVID-19 was 25% ^21^, which is lower than that in our cohort of tracheostomized patients, perhaps due to the low proportion of patients immunized against COVID-19 and longer mechanical ventilation and intensive care stay.

Although the fact that vaccines arrived in Peru in February 2021, vaccination in our region started some months later ^2^ and that the number of COVID-ICU beds was not constant throughout the pandemic ^14^, we could not find statistical differences in relation to deaths and decannulated patients according to the infection period (2020, 2021, 2022), probably because most of our patients were not vaccinated at the time of the study, and the management of these critical patients was always protocolized and homogeneous, since it was always in charge of highly trained human resources in intensive care ^23^.

The benefits of performing an early tracheostomy (≤7 days) in critically ill patients with pneumonia are the reduction of ventilator-associated pneumonia (VAP), more days free of mechanical ventilation, and shorter stay in the ICU ^22^. However, these benefits have not been demonstrated in critically ill patients diagnosed with COVID-19 ^23^, where even higher mortality has been found in those patients with COVID-19 undergoing early tracheostomy (≤ 14 days from orotracheal intubation) compared to late tracheostomy ^16^^,^^24^^,^^25^. In our study the time from intubation to tracheostomy was not associated with mortality, and although most of our patients underwent late tracheostomy, we did not find differences in the incidence of VAP, days of mechanical ventilation, or ICU length of stay, between decannulated and deceased patients. We presume that, due to the complexity of these patients, achieving ventilatory stability was preferred to be able to perform the procedure in most cases.

Usually, tracheostomy is a safe procedure in which the most frequently reported complications are bleeding (7-20%), emphysema (4.4%), stenosis or obstruction (2%) and false airway, with a slightly lower number of complications in percutaneous tracheostomies ^20^^,^^26^^,^^27^. Another alternative is bronchoscopy-guided percutaneous tracheostomy, which allows direct visualization of the airway, increasing the safety of the procedure by allowing the location of the cannulation site, with a low incidence of late complications (<1%) ^28^. And although tracheostomy is a procedure that can cause aerosols and therefore a higher risk of SARS-CoV-2 infection, a study showed that no health worker involved in the tracheostomy procedure developed COVID-19 infection ^9^.

Peak pressure is the pressure reached during inspiration, when air is pushed into the lungs and is a measure of airway resistance ^29^. In the management of the patient with respiratory distress syndrome it is recommended that this value be <25 cm H_2_O to avoid pressure damage (barotrauma) ^30^. We found that this value was higher in patients who died, both in the bivariate analysis and in the crude regression model, although the adjusted regression model did not show a significant value.

Procalcitonin is an indicator of COVID-19 severity, and serial procalcitonin measurements may be useful in predicting disease prognosis ^31^^,^^32^. We found that 85% of patients with a high procalcitonin value died. These patients probably had a more intense hyperinflammatory state due to COVID-19, or bacterial superinfections ^33^^)^ at the time of tracheostomy, which limited their recovery. Likewise, several studies have reported an inversely proportional association between PaO_2_/FiO_2_ and mortality in patients with COVID-19 ^13^^,^^22^^,^^33^^,^^34^. In our study, the median PaO_2_/FiO_2_ at the time of tracheostomy was higher than that reported by other authors, who coincidentally observed higher mortality ^18^, a value less than or equal to 150 mmHg, was the most important factor associated with mortality in our cohort. Due to this finding, it is suggested to avoid tracheostomy when PaO_2_/FiO_2_ lower or equal to 150 mmHg.

Our study has some limitations. The main one was its retrospective nature, which did not allow us to fully evaluate other confounding variables. Another important limitation was the small number of patients, which reduced the possibility of controlling for some confounding factors, and this could also explain why the confidence intervals were so wide and why many variables did not present statistical association, such as the time of tracheostomy (early vs. late), period of infection or exposure to vaccines. Likewise, we cannot affirm whether the elevated procalcitonin level at the time of tracheostomy is due to persistence of COVID-19 or to an added infectious complication and therefore explain the lower survival of this group of patients as in other studies ^35^. Likewise, complications related to the percutaneous tracheostomy procedure in the short term (bleeding, pneumothorax, false airway, etc.) and long term (stenosis, tracheomalacia, fistulas, etc.) were not studied. Finally, it was impossible to measure the impact of changes in treatment strategies (corticosteroids, tocilizumab, etc.) and control methods implemented throughout the pandemic ^20^, which directly affect the practice of tracheostomy.

In conclusion, approximately one third of the patients in this cohort died during the study period. Among the factors associated with higher mortality in tracheostomy patients were a procalcitonin value >0.50ng/dL, and a PaO_2_/FiO_2_ value ≤150 at the time of performing the tracheostomy. Timely recognition of these factors should call for reflection of the personnel performing this procedure, to defer it until oxygen and infectious conditions improve, and thus decrease mortality in this group of critically ill patients.
