# Supplementary material for: Characteristics and factors associated with mortality in tracheostomized patients with COVID-19: a retrospective cohort study in a hospital in Tacna, Peru
Source: Rev Peru Med Exp Salud Publica. 2023 Dec 18;40(4):441–50. doi: 10.17843/rpmesp.2023.404.12629 (PMC11138828; doi:10.17843/rpmesp.2023.404.12629)
Supplement: Translation

# Translation: Características y factores asociados a la mortalidad en pacientes con COVID-19 traqueostomizados: estudio de cohorte retrospectivo en un hospital de Tacna, Perú

**Authors:** Rodrigo Jesús Flores Palacios, Miguel Hueda Zavaleta, Andrés Guillermo Gutiérrez Avila, Juan Carlos Gómez de la Torre, Vicente Aleixandre Benites Zapata

**Affiliations:** 1 Hospital III Daniel Alcides Carrión, Tacna, Perú.; 2 Escuela de Posgrado, Universidad Privada de Tacna, Tacna, Perú.; 3 Diagnóstico, tratamiento e investigación de enfermedades infecciosas y tropicales, Universidad Privada de Tacna, Tacna, Perú.; 4 Laboratorio Clínico Roe, Lima, Perú.; 5 Universidad San Ignacio de Loyola, Unidad de Investigación para la Generación y Síntesis de Evidencias en Salud, Lima, Perú.

**Keywords:** Traqueostomía, Traqueotomía, Supervivencia, SARS-CoV-2, COVID-19, Mortalidad, Perú, Factores de riesgo

## Abstract

**Objetivo.:**

Describir las principales características demográficas, clínicas, laboratoriales y terapéuticas e identificar si están asociados con la mortalidad en pacientes traqueostomizados.

**Material y métodos.:**

Estudio de cohorte retrospectiva en pacientes adultos con diagnóstico de COVID-19, ingresados a UCI (Unidad de Cuidados Intensivos) y que requirieron traqueostomía. Se extrajeron datos demográficos, clínicos, laboratoriales y de tratamiento de las historias clínicas de pacientes que ingresaron al Hospital III Daniel Alcides Carrión de Tacna. Para el análisis de supervivencia se empleó el modelo de riesgos proporcionales de Cox y se calcularon los cocientes de riesgo instantáneos (HR) con sus intervalos de confianza al 95% (IC95%).

**Resultados.:**

Se evaluaron 73 pacientes, el 72,6% eran hombres, las comorbilidades más comunes fueron obesidad (68,5%), diabetes *mellitus* tipo 2 (35,6%) e hipertensión arterial (34,2%). El 37% de los participantes fallecieron durante la estancia en UCI. La mediana de tiempo desde la intubación hasta la traqueostomía y la duración de esta fue 17 (RIC: 15−21) y 21 (RIC: 3−39) días, respectivamente. El análisis multivariado mostró que los factores asociados a mortalidad, fueron presentar un valor de procalcitonina > 0,50 ng/dL en el momento de la traqueostomía (HRa: 2,40 IC95%: 1,03−5,59) y el nivel de PaO_2_/FiO_2_ menor o igual a 150 mmHg, (HRa: 4,44 IC95%: 1,56−12,60).

**Conclusiones.:**

Los factores asociados a mortalidad al momento de realizar la traqueostomía fueron presentar un valor de procalcitonina > 0,50 ng/dL y un cociente PaO_2_/FiO_2_ menor o igual a 150 mmHg.

## INTRODUCCIÓN

La enfermedad por coronavirus 2019 (COVID-19), ha ocasionado más de 5,4 millones de muertes en todo el mundo y es la más importante crisis de salud desde la pandemia de influenza en 1918 ^1^. Hasta el 24 de julio del 2023, en el Perú se diagnosticaron cerca de 4,5 millones de casos de COVID-19 con 221 203 fallecidos y una letalidad del 4,9% ^2^. Perú es el séptimo país con mayor cantidad de fallecidos en el mundo y el tercero en Latinoamérica ^3^.

Los pacientes con síndrome de distrés respiratorio agudo (SDRA) y COVID-19, suelen permanecer intubados durante periodos prolongados ^4^, Cuando el tiempo de intubación y ventilación mecánica supera los 14 días (ventilación mecánica prolongada) la traqueostomía desempeña un rol fundamental en el manejo de estos pacientes, al permitir una mejor gestión de la vía aérea y facilitar la retirada del soporte ventilatorio invasivo. La traqueostomía se ha asociado a menor necesidad de sedación profunda, menor tiempo de destete del ventilador mecánico, menor estancia en la unidad de cuidados intensivos (UCI) y menor estancia hospitalaria ^5^.

Sin embargo, la mayoría de pacientes en UCI candidatos a traqueostomía, tienen mayor riesgo de fallecer, debido a las siguientes características: a) La estancia en la UCI aumenta el riesgo de infecciones nosocomiales ^6^; b) la ventilación mecánica prolongada, que requiere mayor tiempo de sedación, analgesia y relajación muscular, ocasiona polineuropatía y miopatía asociadas a la inmovilidad ^7^, c) el delirio al retiro de la sedoanalgesia; d) el proceso inflamatorio severo (tormenta de citoquinas) provocado por la COVID-19 afecta directamente el intercambio gaseoso ^8^ y e) la edad avanzada y presencia de comorbilidades que suelen presentar los pacientes admitidos en la UCI ^9^.

Asimismo, la traqueostomía es un procedimiento que no está exento de riesgos y complicaciones potenciales como hemorragia, infección del sitio quirúrgico, neumotórax y estenosis traqueal. Todas estas condiciones hacen que la realización de la traqueostomia no sea siempre segura ^4^. Por lo tanto, aunque un paciente con COVID-19 grave tenga la indicación de una traqueostomia, se debe identificar aquellos factores asociados a mayor mortalidad, para mejorar la toma de decisiones clínicas y optimizar los resultados, teniendo en cuenta la alta complejidad de estos y la mortalidad ya acumulada por las propias condiciones basales de estos pacientes.

Hasta el momento los factores relacionados a la mortalidad en los pacientes críticamente enfermos traqueostomizados por COVID-19, son el nivel de proteína C reactiva (PCR) elevado en el momento de la traqueostomia ^10^, mayor requerimiento de fracción inspirada de oxígeno (FiO_2_) ^11^ y el momento temprano de realización ^12^. Lamentablemente, la mayoría de esta información proviene de países desarrollados, y no refleja la realidad de Perú, país que tiene un frágil sistema de salud. En este contexto, la enfermedad crítica por COVID-19 fue más frecuente, agresiva y mortal ^13^, ya que luego de las tres olas de esta enfermedad, hubo un aumento de pacientes críticos respiratorios y de pacientes traqueostomizados, con una mortalidad del 32,9% en nuestra UCI ^14^.

El objetivo de este trabajo es describir las características demográficas, clínicas, laboratoriales, terapéuticas e identificar los predictores que permitan identificar oportunamente los pacientes con COVID-19 que tienen mayor riesgo de fallecer luego de una traqueostomía percutánea, ya que este grupo de pacientes constituyen una población con estancia prolongada y alta mortalidad ^15^.

**MENSAJE CLAVE**

Motivación para realizar el estudio: Los pacientes con diagnóstico de COVID-19 que son candidatos a la realización de una traqueostomía, son pacientes complejos con larga estancia hospitalaria y alto riesgo de muerte, la realización de este procedimiento médico en las mejores condiciones posibles resulta fundamental para su supervivencia.

Principales hallazgos: Los factores de riesgo de fallecer al momento de realizar una traqueostomía fueron presentar niveles bajos del cociente PaO_2_/FiO_2_ (menor o igual a 150) y un nivel de procalcitonina >0,5 ng/mL.

Implicancias: El reconocimiento temprano de estos factores de riesgo, pueden ayudar a encontrar el mejor momento del paciente para realizar la traqueostomía de manera más segura y con menores complicaciones.

## MATERIALES Y MÉTODOS

### Diseño del estudio

Se realizó un estudio de tipo cohorte retrospectiva, los datos se recolectaron entre septiembre y octubre del 2022 e incluyó a los pacientes con COVID-19 crítico, definido por la presencia de SDRA, sepsis o shock séptico ^16^; que estuvieron hospitalizados en el Hospital Daniel Alcides Carrion de Tacna, desde el 28 de marzo del 2020 al 1 de marzo del 2022.

### Población

Se analizaron 329 historias clínicas obtenidas del Servicio de Salud Inteligente (ESSI), el cual es un software donde se encuentran las historias clínicas en formato digital ^17^, que incluyen a la totalidad de pacientes hospitalizados en la UCI de COVID-19 (UCI-COVID-19) del Hospital Daniel Alcides Carrión - EsSalud, nivel III ubicado en Tacna, Perú, que tiene más de 120 camas para pacientes con COVID-19 y 14 camas en la UCI-COVID ^14^. Los pacientes críticos fueron tratados de acuerdo con los protocolos institucionales elaborados para pacientes con COVID-19 grave; estos protocolos se actualizan de acuerdo con la nueva evidencia científica.

Se incluyeron pacientes mayores de 18 años, admitidos en la UCI-COVID, con diagnóstico confirmado de infección por el SARS-CoV-2, insuficiencia respiratoria grave por COVID-19 y necesidad de ventilación mecánica invasiva que, durante su estancia hospitalaria, requirieron la colocación de una traqueostomía percutánea. Todas las traqueostomías fueron realizadas de forma percutánea, por dos médicos de la UCI, uno encargado de la vía aérea y el otro encargado del procedimiento propiamente dicho. Se excluyeron a pacientes que ingresaron a la UCI, por motivo diferente de neumonía por COVID-19, pacientes con datos clínicos/laboratoriales incompletos y pacientes cuyo desenlace no fue muerte o decanulación. La muerte del paciente se corroboró a través del ESSI.

Si bien se analizaron 329 historias clínicas, solo se incluyeron 73 historias clínicas de pacientes traqueostomizados. La potencia estadística se calculó en base al estudio de Tang *et al*. ^26^. donde el 73,3% de los pacientes con traqueostomía temprana (≤14 días) murieron durante el seguimiento, en comparación con el 42% de mortalidad en aquellos con traqueostomía tardía (>14 días). Asimismo, se consideró una razón de no expuestos/expuestos de 1,30 (73/56). Con estos parámetros, con un nivel de confianza del 95% y 73 participantes de nuestra muestra se obtuvo un poder estadístico de 76,5%.

### Variables de estudio

Se consideró como variable dependiente, la muerte durante la hospitalización (si/no), siendo los sobrevivientes los pacientes que fueron decanulados y posteriormente dados de alta, esta información se recolecto directamente del ESSI. Las variables independientes fueron la edad (años), el sexo (masculino/femenino). Las comorbilidades se consideraron variables dicotómicas (sí/no) y fueron obesidad, diabetes *mellitus*, hipertensión arterial, y enfermedad renal crónica. El periodo de infección se consideró por años: 2020, 2021 y 2022. La vacunación (2 dosis /3 dosis). Las variables temporales que se consideraron fueron: estancia hospitalaria (días), estancia en UCI (días), días de ventilación mecánica, días desde la intubación hasta la traqueostomia, días de traqueostomia y se categorizó a la traqueostomia tardía como >14dias (sí/no).

Los datos de laboratorio fueron leucocitos (>12 000 células/mm^3^), proteína C reactiva (PCR>6 mg/dL), procalcitonina (procalcitonina> 0,5 ng/dL), PaO_2_/FiO_2_ (presión arterial de oxígeno / fracción inspirada de oxígeno) al momento de la traqueostomia (≤150 mmHg), creatinina (>1,3 mg/dL). Las variables ventilatorias fueron: FiO_2_ (>35%), PEEP (presión positiva al final de la espiración) (>8 cm H_2_O), volumen tidal (>460 mL ) y presión Inspiratoria (15 cm H_2_O), presión pico (>25 cm H_2_O), frecuencia respiratoria (>25 rpm). Las complicaciones durante la hospitalización fueron: sobreinfecciones, definidas como deterioro clínico registrado y asociado a un cultivo positivo (si/no), estas fueron: bacteriemia (sí/no), neumonía asociada a ventilador (sí/no), infección del tracto urinario (sí/no), shock séptico (sí/no) y barotrauma (sí/no).

### Procedimientos

Se incluyeron a todos los pacientes hospitalizados durante la pandemia de COVID-19, desde el 28 de marzo del 2020 al 1 de marzo del 2022. Una vez identificados se procedió a la revisión de historias clínicas a través del ESSI, se recabó la información de sus comorbilidades, diagnósticos, exámenes de laboratorio, procedimientos y desenlace.

La información fue recolectada por los investigadores del estudio (RFP, MHZ y AGA) en una hoja de cálculo creada en Microsoft Excel 2019, se hizo doble digitación para el control de inconsistencias, se detallaron características demográficas, clínicas y laboratoriales, al momento de la realización de la traqueostomía y adicionalmente también se recopiló la terapéutica instaurada y su desenlace durante la hospitalización (decanulación o muerte).

El seguimiento de los casos se hizo durante su estancia en cuidados críticos, se consideró tiempo cero el momento de la traqueostomía y el tiempo final a la ocurrencia de la muerte o alta de la unidad de cuidados intensivos en los pacientes decanulados.

### Análisis estadístico

Los datos se importaron al programa Stata® v17 (StataCorp., College Station, TX, EE.UU.) Las comparaciones se realizaron entre los pacientes críticamente enfermos portadores de traqueostomia, que lograron ser decanulados con éxito y los que fallecieron durante su estancia en la UCI-COVID. Las variables categóricas se expresaron como frecuencias y porcentajes, y las variables categóricas numéricas como medianas y rangos intercuartílicos, según corresponda. Todas las pruebas de hipótesis fueron de dos colas, con un nivel de significancia de 0,05. Para la comparación de variables se empleó la prueba chi cuadrado o la prueba exacta de Fisher (según corresponda) y la prueba U-Mann-Whitney en las variables numéricas, debido a su distribución no normal. Para determinar los factores asociados a mortalidad al momento de realizar la traqueostomia, se usó el modelo de riesgos proporcionales de Cox, teniendo como variable temporal a los días transcurridos desde la hospitalización hasta la muerte o alta (en los decanulados), para identificar los cocientes de riesgos instantáneos (HR) crudos y ajustados y sus respectivos intervalos de confianza (IC) del 95%.

Las variables que ingresaron al análisis crudo fueron aquellas que mostraron asociación estadísticamente significativa en el análisis bivariado de mortalidad (p<0,05). Las variables que ingresaron al análisis ajustado fueron aquellas que mostraron asociación estadísticamente significativa con mortalidad en el análisis crudo (p<0,05). Se corroboraron los supuestos de proporcionalidad mediante residuos de Schoenfeld. La colinealidad de las variables ingresadas a la regresión de Cox fueron calculadas mediante el factor de inflación de la varianza (VIF) teniendo como punto de corte 6.0. Todos los valores de las variables incluidas en la regresión fueron aceptables [periodo de infección (VIF=3,06), procalcitonina>0,5ng/dL (VIF=1,73), PaO_2_/FiO_2_ <150 mmHg (VIF=2,21), FiO_2_ >35% (VIF=1,98), y presión pico >25 (VIF=3,33)].

Finalmente, describimos la supervivencia de los pacientes sometidos a traqueostomia admitidos por COVID-19 mediante el método de Kaplan-Meier. Se utilizó la prueba de log-rank para evaluar las diferencias entre las funciones de supervivencia.

### Aspectos éticos

El presenta trabajo de investigación está suscrito a las normas de Helsinki para la investigación en seres humanos. Se obtuvo la aprobación del protocolo de investigación por parte del comité de ética del Hospital III Daniel Alcides Carrión (CIEI-Tacna-N.º 036). Se registro el trabajo de investigación en la plataforma de Proyectos de Investigación en Salud (PRISA) del Instituto Nacional de Salud con el código EI00000003060. Debido a la naturaleza retrospectiva y observacional del estudio, no se solicitó consentimiento informado, además se guardó la confidencialidad de los datos escrutados.

## RESULTADOS

De los 329 pacientes críticos hospitalizados en la UCI-COVID-19, a 83 (25,2%) se les realizo traqueostomía como parte de su evolución, pero 10 no cumplieron los criterios de inclusión (un paciente ingreso con diagnostico diferente a neumonía por COVID-19, 2 pacientes no tuvieron el desenlace de muerte o decanulación y 7 pacientes tenían datos clínicos y de laboratorios incompletos), se incluyó un total de 73 historias clínicas de pacientes admitidos en UCI-COVID con diagnóstico de infección por SARS-CoV-2, neumonía por COVID-19 y portadores de traqueostomía percutánea.

La mediana de edad fue 59 (RIC: 52−66) años, el 72,6% fueron varones y solo el 6,8% estaban vacunados contra el SARS-CoV-2. Las comorbilidades más frecuentes fueron obesidad (68,4%), diabetes *mellitus* tipo 2 (35,6%) e hipertensión arterial (34,2%).

La mediana de tiempo de estancia en UCI y de ventilación mecánica (VM) fue de 32 (RIC: 24-41) y 31 (RIC: 24-43) días, respectivamente, la mediana de tiempo de permanencia de la traqueostomía fue de 18 (RIC: 10-25) días (decanulación o muerte) y el momento de realización de la traqueostomía fue a los 17 (RIC: 15-20) días contados desde el momento de la intubación.

En relación con los análisis de laboratorio, la mediana de PaO_2_/FiO_2_ en el momento de la traqueostomía fue 213 (RIC: 175-270), procalcitonina en 0,22 ng/mL (RIC: 0,13-0,40), PCR de 6,35 mg/dL (RIC: 3,2-12,1), leucocitos de 11,950 cel/mL (8,810-15,510). En cuanto a las variables de la ventilación mecánica, el FiO_2_ al momento de la traqueostomía fue de 30% (RIC: 28-35), la PEEP (presión positiva al final de la espiración) fue 7 cm H_2_O (RIC: 6-8), la presión inspiratoria de 15 cm H_2_O (RIC: 14-18) y el volumen tidal de 466 mL (RIC: 406-509).

En cuanto al tratamiento, 71 (97,3%) pacientes recibían antibióticos, 20 (27,4%) antifúngicos y 9 (12,3%) vasopresores en el momento de realización de la traqueostomía. Las complicaciones infecciosas fueron: neumonía asociada a VM en 66 pacientes (90,4%), bacteriemia 14 (19,2%) e infección del tracto urinario en 9 (12,3%) pacientes. Durante la estancia en UCI, 27 (37%) pacientes traqueostomizados fallecieron; y 46 (63%) fueron decanulados con éxito y fueron dados de alta (Tabla 1).

**Tabla 1:** Características clínicas, de laboratorio y parámetros ventilatorios de la población de estudio y comparación entre decanulados sobrevivientes y fallecidos.

Variable	Total	Sobrevivientes	Fallecidos	Valor de p
	n=73 (%)	decanulados	n=27 (%)	
		n=46 (%)		
Características demográficas				
Edad ^a^	59 (52-66)	58,5 (49-65)	61 (52-66)	0,212^b^
Sexo				
Masculino	53 (72,6)	34 (64,2)	19 (35,8)	0,743^c^
Femenino	20 (27,4)	12 (60,0)	8 (40,0)	
Características clínicas				
Comorbilidades				
Sí	65 (89,0)	39 (60,0)	26 (40,0)	0,247^c^
No	8 (11,0)	7 (87,5)	1 (12,5)	
Obesidad				
Sí	50 (68,5)	20 (58,0)	21 (42,0)	0,191^c^
No	23 (31,5)	17 (73,9)	6 (26,1)	
Diabetes *mellitus*				
Sí	26 (35,6)	15 (57,7)	11 (42,3)	0,484^c^
No	47 (64,4)	31 (65,9)	16 (34,1)	
Hipertensión				
Sí	25 (34,3)	15 (60,0)	10 (40,0)	0,700^c^
No	48 (65,7)	31 (64,6)	17 (35,4)	
Enfermedad renal crónica				
Sí	3 (4,1)	2 (66,7)	1 (33,3)	0,693^d^
No	70 (95,9)	44 (62,8)	26 (37,2)	
Periodo de infección				
2020	22 (30,1)	10 (21,7)	12 (44,4)	
2021	42 (57,5)	32 (69,6)	10 (37,0)	0,025^c^
2022	9 (12,4)	4 (8,7)	5 (18,6)	0,261^d^
Vacunación contra SARS-CoV-2	5 (6,9)	2 (40,0)	3 (60,0)	
2 dosís	2 (2,7)	0 (0,0)	2 (100,0)	
3 dosís	3 (4,1)	2 (66,7)	1 (33,3)	
Días de estancia hospitalaria ^a^	41 (34-51)	47,5 (39-57)	34 (29-40)	<0,001^b^
Días de estancia en UCI ^a^	32 (24-41)	32,5 (24-46)	27 (22-36)	0,172^b^
Días en ventilación mecánica ^a^	31 (24-43)	31,5 (23-45)	31 (27-38)	0,899^b^
Días desde intubación hasta traqueostomía ^a^	17 (15-20)	16 (13-21)	17 (15-20)	0,791^b^
Días de traqueostomía ^a^	18 (10-25)	19 (10,5-27,5)	13 (8-20)	0,087^b^
Traqueostomía tardía >14 días				
Sí	56 (76,7)	33 (58,9)	23 (41,1)	0,189^c^
No	17 (23,3)	13 (76,5)	4 (23,5)	
Características laboratoriales al momento de Traqueostomía				
Leucocitos >12000 cel/mm^3^				
Sí	36 (49,3)	19 (52,8)	17 (47,2)	0,074^c^
No	37 (50,7)	27 (73,0)	10 (27,0)	
Proteína C reactiva >6 mg/dL				
Sí	33 (45,2)	17 (51,5)	16 (48,5)	0,065^c^
No	40 (54,8)	29 (72,5)	11 (27,5)	
Procalcitonina >0,5 ng/dL				
Sí	14 (19,2)	2 (14,3)	12 (85,7)	<0,001^c^
No	59 (80,8)	44 (74,6)	15 (25,4)	
PaO_2_/FiO_2_ <150 mmHg				
Sí	9 (12,3)	0 (0,0)	9 (100,0)	<0,001^c^
No	64 (87,7)	46 (71,8)	18 (28,2)	
Creatinina >1,3 mg/dL				
Sí	9 (12,3)	5 (55,6)	4 (44,4)	0,621^c^
No	64 (87,7)	41 (64,0)	23 (36,0)	
Parámetros ventilatorios al momento de traqueostomía				
FiO_2_ >35%				
Sí	27 (37,0)	11 (40,7)	16 (59,3)	0,003^c^
No	46 (63,0)	35 (76,1)	11 (23,9)	0,003^c^
PEEP >8 cm H_2_O				
Sí	28 (38,4)	16 (57,1)	12 (42,9)	0,412^c^
No	45 (61,6)	30 (66,7)	15 (33,3)	
Volumen tidal >460 mL				
Sí	41 (56,2)	27 (65,9)	14 (34,1)	0,569^c^
No	32 (43,8)	19 (59,3)	13 (40,7)	
Presión inspiratoria >15 cm H_2_O				
Sí	48 (65,8)	28 (58,3)	20 (41,7)	0,251^c^
No	25 (34,2)	18 (72,0)	7 (28,0)	
Presión pico >25 cm H_2_O				
Sí	28 (38,4)	12 (42,9)	16 (57,1)	0,005^b^
No	45 (61,6)	34 (75,5)	11 (24,5)	
Frecuencia respiratoria >25rpm				
Sí	22 (30,1)	13 (59,1)	9 (40,9)	0,648^c^
No	51 (69,9)	33 (64,7)	18 (35,3)	
Complicaciones				
Infección nosocomial				
Sí	64 (87,7)	39 (60,9)	25 (39,1)	0,701^d^
No	9 (12,3)	7 (77,7)	2 (33,3)	
Neumonía asociada a ventilador				
Sí	66 (90,4)	41 (62,1)	25 (37,9)	0,483^d^
No	7 (9,6)	5 (71,4)	2 (28,6)	
Bacteriemia				
Sí	14 (19,2)	10 (71,4)	4 (28,6)	0,344^d^
No	59 (80,8)	36 (61,0)	23 (39,0)	
ITU				
Sí	9 (12,3)	5 (55,6)	4 (44,4)	0,440^d^
No	64 (87,7)	41 (64,0)	23 (36,0)	
Shock séptico				
Sí	63 (86,3)	42 (66,7)	21 (33,3)	0,098^d^
No	10 (13,7)	4 (40,0)	6 (60,0)	
Barotrauma				
Sí	14 (19,2)	7 (50,0)	7 (50,0)	0,262^c^
No	59 (80,8)	39 (66,1)	20 (33,9)	0,262^c^

a Mediana y rango intercuartílico, ^b^ prueba U de Mann-Whitney, ^c^ prueba chi cuadrado, ^d^ prueba exacta de Fisher.

UCI: unidad de cuidados intensivos, PaO_2_/FiO_2_: relación presión arterial de oxígeno y fracción inspirada de oxígeno (FiO_2_), PEEP: presión positiva al final de la espiración, NAV: neumonía asociada a ventilador, ITU: infección del tacto urinario, rpm: respiraciones por minuto.

No se observaron diferencias significativas respecto a la mortalidad en pacientes según grupo etario, sexo, estado de vacunación, comorbilidades, tiempo de estancia en UCI, tiempo en ventilación mecánica, momento de la traqueostomía, ni según los tratamientos recibidos. Sin embargo, el tiempo de permanencia de la traqueostomía, fue superior en los que lograron su decanulación, con respecto a los que fallecieron (21 *vs.* 15 días; p=0,043). Asimismo, La PaO_2_/FiO_2_ fue menor en el grupo de pacientes que fallecieron (p<0,001), mientras que un valor de procalcitonina >0,5mg/dL, mayor valor de FiO_2_, presión inspiratoria y presión pico al momento de la traqueostomía fueron asociados con mayor riesgo de fallecer.

Los factores asociados a mortalidad se evaluaron mediante el análisis de regresión de riesgos proporcionales de Cox. En el análisis ajustado, la procalcitonina >0,50 en el momento de la traqueostomía se mostró como un factor asociado a mortalidad con un HRa de 2,40 (IC 95%: 1,03-2,59). Asimismo, el nivel de PaO_2_/FiO_2_ ≤150 mmHg se asoció de forma independiente con mayor mortalidad, presentando un HRa de 4,40 (IC 95%: 1,56-12,60) (Tabla 2). La figura 1 presenta las curvas de supervivencia de Kaplan-Meier para las variables PaO_2_/FiO_2_ (p<0,001) y procalcitonina (p<0,001).

**Tabla 2:** Análisis de regresión de Cox para evaluar predictores de mortalidad en pacientes traqueostomizados hospitalizados por COVID-19.

Variable	HRc (IC95%)	Valor de p	HRa (IC95%)	Valor de p
Periodo de infección				
2020	Ref.		-	
2021	0,52 (0,22-1,21)	0,130	-	
2022	1,13 (0,39-3,26)	0,816	-	
Procalcitonina >0,5 ng/dL				
Sí	3,76 (1,75-8,10)	0,001	2,41 (1,04-2,60)	0,005
No	Ref.		Ref.	
PaO_2_/FiO_2_ ≤150 mmHg				
Sí	7,60 (3,21-17,99)	<0,001	4,41 (1,57-12,61)	0,005
No	Ref.		Ref.	
FiO_2_ >35%				
Sí	2,02 (0,93-4,36)	0,075	-	-
No	Ref.		-	
Presión pico >25 cmH_2_0				
Sí	2,40 (1,09-5,29)	0,030	1,31 (0,52-3,30)	0,569
No	Ref.		Ref.	

HRc: hazard ratio crudo; HRa: hazard ratio ajustado; PaO_2_/FiO_2_: relación presión arterial de oxígeno y fracción inspirada de oxígeno (FiO_2_); PEEP: presión positiva al final de la espiración. Las variables que ingresaron al análisis ajustado, fueron aquellas que mostraron asociación estadísticamente significativa con mortalidad en el análisis crudo (p<0,05).

**Figura 1:** Curvas de supervivencia de Kaplan-Meier según la categoría de nivel de PaO2/FiO2 y procalcitonina, censurados a los 30 días.

## DISCUSIÓN

En el presente estudio falleció el 36,9% de todos los pacientes que fueron traqueostomizados por COVID-19, Además que pudimos establecer dos factores de riesgo para mortalidad, que son el valor de procalcitonina >0,5 ng/dL, y los niveles bajos del cociente PaO_2_/FiO_2_ (menor o igual a 150) al momento de la realización de la traqueostomía.

La mortalidad reportada en pacientes con traqueostomía y COVID-19, oscila entre 15 a 66% ^18^^-^^21^. Un metaanálisis de 5268 pacientes traqueostomizados reportó una mortalidad agrupada de 22%, sin embargo, se observó gran heterogeneidad entre los estudios incluidos ^19^. También se ha descrito que los pacientes críticos sometidos a traqueostomía presentaron menor mortalidad que aquellos no traqueostomizados, incluso en pacientes severamente enfermos con APACHE II >17 ^18^^,^^21^. En Perú, la mortalidad global por SDRA asociada a COVID-19 fue de 25% ^21^, lo cual es inferior a la observada en nuestra cohorte de traqueostomizados, quizás por la baja proporción de inmunizados contra la COVID-19 y mayor tiempo de ventilación mecánica y estancia en cuidados intensivos.

Pese que, en el Perú las vacunas llegaron en febrero del 2021, la vacunación en nuestra región inicio algunos meses después ^2^ y además la cantidad de camas de UCI-COVID no fueron constantes a lo largo de la pandemia ^14^, no pudimos encontrar diferencias estadística en relación a los fallecidos y decanulados según periodo de infección (2020,2021,2022), probablemente debido a que la mayoría de nuestros pacientes no estaban vacunados al momento del estudio, ademas el manejo de estos pacientes críticos siempre fue protocolizado y homogéneo, ya que estuvo siempre a cargo de recurso humano altamente capacitado en terapia intensiva ^23^.

Los beneficios de la realización de una traqueostomia temprana (≤7dias) en pacientes con neumonía en estado crítico son: disminución de la neumonía asociada a ventilador (NAV), más días libres de ventilación mecánica, y menor estancia en UCI ^22^. Sin embargo, estos beneficios no han sido demostrados en pacientes críticos con diagnóstico de COVID-19 ^23^, donde inclusive se ha encontrado mayor mortalidad en aquellos pacientes con COVID-19 sometidos a una traqueostomía temprana (≤ 14 días desde la intubación orotraqueal) comparado a una traqueostomía tardía ^16^^,^^24^^,^^25^. En nuestro estudio el tiempo desde la intubación hasta la traqueostomía no fue una variable asociada a mortalidad, y aunque la mayoría de nuestros pacientes fueron sometidos a una traqueostomía tardía, no observamos diferencias en la incidencia de NAV, días de ventilación mecánica, ni en el tiempo de estancia en UCI, entre los pacientes decanulados y los fallecidos. Presumimos que, debido a la complejidad de estos pacientes, se prefería alcanzar la estabilidad ventilatoria para poder realizar el procedimiento en la mayoría de los casos.

Por lo general, la traqueostomía es un procedimiento seguro donde las complicaciones más frecuentemente reportadas fueron sangrado (7-20%), enfisema (4,4%), estenosis u obstrucción (2%) y falsa vía aérea, con un discreto menor número de complicaciones en las traqueostomías percutáneas ^20^^,^^26^^,^^27^. Otra alternativa es la traqueostomía percutánea guiada por broncoscopía, la cual permite la visualización directa de las vías respiratorias, aumentando la seguridad del procedimiento al permitir la ubicación del sitio de canulación, con baja incidencia de complicaciones tardías (<1%) ^28^. Y aunque la traqueostomía es un procedimiento que puede generar aerosoles y por tanto mayor riesgo de contagio por SARS-CoV-2, en un estudio ningún trabajador de salud involucrado en el procedimiento de traqueostomía desarrollo infección por COVID-19 ^9^.

La presión pico es la presión alcanzada durante la inspiración cuando el aire es empujado hacia los pulmones y es una medida de la resistencia de las vías respiratorias ^29^. En el manejo del paciente con síndrome de distres respiratorio se recomienda que este valor sea <25 cm H_2_O para evitar el daño por presión (barotrauma) ^30^. En nuestro estudio observamos que este valor fue más alto en los pacientes que fallecieron, tanto en el análisis bivariado, como en el modelo de regresión cruda, aunque en el modelo de regresión ajustado no alcanzo un valor significativo.

El nivel de procalcitonina es un indicador de gravedad de la COVID-19 y las mediciones seriadas de procalcitonina pueden ser útiles para predecir el pronóstico de enfermedad ^31^^,^^32^. En nuestro estudio, observamos que el 85% de pacientes con un valor elevado de procalcitonina fallecieron. Probablemente estos pacientes cursaban con un estado hiperinflamatorio más intenso por la COVID-19, o sobreinfecciones bacterianas ^33^ al momento de la traqueostomía, que limitaron la recuperación de estos casos. Asimismo, diversos estudios han observado una asociación inversamente proporcional entre la PaO_2_/FiO_2_ y mortalidad en pacientes con la COVID-19 ^13^^,^^22^^,^^33^^,^^34^. En nuestro estudio, la mediana de PaO_2_/FiO_2_ en el momento de la traqueostomia fue superior a lo reportado por otros autores, que coincidentemente observaron mayor mortalidad ^18^, un valor inferior o igual a 150 mmHg, fue el factor más importante asociado a mortalidad en nuestra cohorte. Debido a este hallazgo, se sugiere evitar la traqueostomía cuando el PaO_2_/FiO_2_ inferior o igual a 150 mmHg.

Nuestro estudio tiene algunas limitaciones. La principal fue su naturaleza retrospectiva, que no permitió evaluar completamente otras variables confusoras. Otra limitación importante fue el número pequeño de pacientes, lo que redujo la posibilidad de controlar algunos factores de confusión, además esto podría explicar que los intervalos de confianza fueran tan amplios y que muchas variables no presentaron asociación estadística, como el momento de la traqueostomia (temprana *vs.* tardía), periodo de infección o la exposición a vacunas. Del mismo modo, no podemos afirmar si el nivel elevado de procalcitonina en el momento de la traqueostomía sea por persistencia del COVID19 o por una complicación infecciosa agregada y por lo tanto explicar la menor sobrevida de este grupo de pacientes como en otros trabajos ^35^. Asimismo, no se midieron las complicaciones relacionadas al procedimiento de traqueostomia percutánea en el corto plazo (hemorragias, neumotórax, falsa vía, etc.) ni largo plazo (estenosis, traqueomalacia, fistulas, etc.). Finalmente fue imposible medir el impacto del cambio en las estrategias de tratamiento (corticoides, tocilizumab, etc.) y los métodos de control implementados a lo largo de la pandemia ^20^, que afectan directamente la práctica de la traqueostomia.

En conclusión, aproximadamente un tercio de los pacientes de esta cohorte fallecieron durante el periodo de estudio. Entre los factores asociados a mayor mortalidad en los pacientes con traqueostomia fueron el valor de procalcitonina >0,50ng/dL, y un valor de PaO_2_/FiO_2_ ≤150 al momento de realizar la traqueostomía. El reconocimiento oportuno de estos factores debe llamar a la reflexión al personal que realiza este procedimiento, para diferirlo hasta que las condiciones oxigenatorias e infecciosas mejoren, y así disminuir la mortalidad en este grupo de pacientes críticamente enfermos.
